# Successes and Challenges of PARP Inhibitors in Cancer Therapy

**DOI:** 10.3389/fonc.2015.00222

**Published:** 2015-10-14

**Authors:** Tiffany K. Ricks, Haw-Jyh Chiu, Gwynn Ison, Geoffrey Kim, Amy E. McKee, Paul Kluetz, Richard Pazdur

**Affiliations:** ^1^Office of Hematology and Oncology Products (OHOP), Office of New Drugs (OND), Center for Drug Evaluation and Research (CDER), U.S. Food and Drug Administration, Silver Spring, MD, USA

**Keywords:** PARP inhibitor, cancer, drug resistance, BRCA1, BRCA2, homologous recombination

## Introduction

Poly (ADP-ribose) polymerases (PARPs) are a family of enzymes involved in cellular homeostasis, including DNA transcription, cell-cycle regulation, and DNA repair (1, 2). PARPs can detect DNA damage and bind to DNA single strand breaks (SSBs) through their N-terminal zinc finger domains. DNA binding activates the C-terminal catalytic domain, which hydrolyzes NAD^+^ to attach poly ADP-ribose (PAR) polymers covalently to nuclear proteins, including PARP itself. Negatively charged PAR polymers promote recruitment of DNA repair proteins, and auto-PARylation causes dissociation of PARPs from DNA, allowing completion of DNA repair. In the absence of PARP activity, unrepaired SSBs can lead to more deleterious double strand breaks (DSBs), which require high fidelity, homologous recombination (HR) or low fidelity, non-homologous end joining (NHEJ) for repair.

*In vitro* and *in vivo* studies have demonstrated that tumor cells harboring defects in DNA repair are highly sensitive to PARP inhibitors, leading to genomic instability and cell death. Two publications demonstrated the concept of synthetic lethality in BRCA-deficient cells treated with PARP inhibitors (3, 4). Cells lacking functional alleles of *BRCA* are defective in HR repair and have an increased susceptibility to cause tumor development. Loss of BRCA or inhibition of PARP alone has little effect on *in vitro* and *in vivo* tumor growth; however, loss of function of both proteins enhances anti-tumor activity. Restoring BRCA expression blocks the cytotoxic effects of PARP inhibitor treatment.

Several clinical PARP inhibitors are under investigation in Phase 2 and Phase 3 clinical trials as monotherapy in cancers with DNA repair defects or in combination with radiation, chemotherapy, or other targeted agents (Table 1). Progress in PARP inhibitor development has led to the recent accelerated approval of Lynparza (olaparib) by the U.S. Food and Drug Administration (5). Lynparza is currently indicated as monotherapy for patients with advanced germline *BRCA*-mutated ovarian cancer who have received three or more prior lines of chemotherapy. Lynparza was approved with a companion diagnostic test to select patients with deleterious or suspected deleterious *BRCA* mutations. PARP inhibitors are anticipated to have a much broader clinical application in additional tumor types, particularly those with DNA repair defects and in combination with chemotherapy and other targeted agents. In light of renewed interest in PARP inhibitors and the recent approval of Lynparza, this review will highlight data of PARP inhibitors in *in vitro* and *in vivo* cancer models and explore some of the clinical applications and challenges of PARP inhibitor therapy.

**Table 1:** **PARP inhibitors in Phase 2 and Phase 3 clinical development^a^**.

PARP inhibitor	Clinical trial	Therapy	Tumor type
Olaparib	Phase 2	Monotherapy	Ovarian, peritoneal, fallopian tube, breast, colorectal, lung, Ewing’s sarcoma, prostate, pancreatic, advanced tumors
		Combination	Breast, ovarian, peritoneal, fallopian tube, endometrial, gastric, prostate, lung, pancreatic
	Phase 3	Monotherapy	Breast, ovarian
		Combination	Ovarian, peritoneal, fallopian tube, gastric
		Maintenance	Ovarian, pancreatic
Veliparib	Phase 2	Monotherapy	Ovarian, fallopian tube, peritoneal, breast
		Combination	Breast, ovarian, peritoneal, fallopian tube, colorectal, lung, cervical, prostate, liver, glioblastoma, melanoma, pancreatic, advanced tumors
	Phase 3	Combination	Breast, lung, glioblastoma
Rucaparib	Phase 2	Monotherapy	Breast, ovarian, fallopian, peritoneal, pancreatic
	Phase 3	Combination	Breast
		Maintenance	Ovarian, fallopian tube, peritoneal
Niraparib	Phase 2	Monotherapy	Ovarian
	Phase 3	Monotherapy	Breast
		Maintenance	Ovarian
Talazoparib	Phase 2	Monotherapy	Breast, ovarian, endometrial, advanced tumors
	Phase 3	Monotherapy	Breast
E7016	Phase 2	Combination	Melanoma

*^a^Completed and active clinical trials obtained from www.clinicaltrials.gov, data accessed August 2015*.

## Mechanisms of Anti-Tumor Effect of Parp Inhibitors

Poly (ADP-ribose) polymerase inhibitors are structurally similar in that they contain a nicotinamide moiety and mimic the NAD^+^ substrate. PARP inhibitors competitively bind to the catalytic domain of PARPs and inhibit PAR synthesis with half-maximal inhibitory concentration (IC_50_) values in the low nanomolar range (6–8). PARP inhibitors were developed to block the enzymatic activity of PARPs and prevent SSB repair by inhibiting the base excision repair (BER) pathway, and initial clinical development focused on potentiating the effects of chemotherapy and radiation (6, 9, 10). Subsequent studies demonstrated that PARP inhibitors alone were cytotoxic in HR-deficient cells (3, 4, 11). Based on these findings, a model was proposed in which PARP inhibition causes unrepaired SSBs, which are subsequently converted to DSBs, leading to synthetic lethality in HR-deficient cells (4). However, knockdown of XRCC1, the protein immediately downstream of PARP in the BER pathway did not lead to synthetic lethality (12), suggesting that loss of PARP activity is critical for synthetic lethality, but the loss of BER is not.

Poly (ADP-ribose) polymerases function in other aspects of DNA repair, and emerging data suggest other mechanisms of action for the anti-tumor activity of PARP inhibitors in HR-deficient cells (13, 14). One potential mechanism proposes that PARP inhibition activates NHEJ in HR-deficient cells, leading to genomic instability and cell death (12). *In vitro* studies have demonstrated that PARPs can regulate components of the NHEJ machinery, including DNA-dependent protein kinase (DNA-PK), Ku70, and Ku80 (15–18). In HR-deficient cells, PARP inhibitor treatment induced the activation of DNA-PK and phosphorylation of downstream substrates and increased NHEJ of a reporter plasmid containing a DSB (12). Pharmacological blockade or loss of NHEJ proteins reduced chromosomal aberrations and the cytotoxic effects of PARP inhibition, indicating a role for NHEJ in PARP inhibitor activity.

*In vitro* studies have demonstrated that the activity of PARP inhibitors may also involve formation of deleterious PARP–DNA complexes, which hinder DNA replication and repair (19–21). Avian cells lacking PARP1 and PARP2 were resistant to olaparib treatment and remained viable at concentrations greater than 10 μM (19). In contrast, olaparib caused significant cytotoxicity in wild type cells and increased levels of γ-H2AX, a marker of DNA damage. PAR polymers were undetectable by ELISA in both olaparib-treated wild type cells and PARP-deficient cells, suggesting that PARP inhibition is distinct from genetic deletion of PARP.

A comparison of PARP inhibitors demonstrated comparable inhibition of PAR synthesis by Western blot and ELISA (19, 20). In contrast, each PARP inhibitor showed varying ability to induce PARP–DNA complexes in the presence of alkylating agent. In the absence of PARP inhibitor, PARP1 was detected in the nuclear soluble fraction by Western blot and accumulated in the chromatin-bound fraction following PARP inhibitor treatment. In tumor cells, BMN 673 (talazoparib) induced greater accumulation of PARP1 and PARP2 in the chromatin-bound fraction compared to olaparib and rucaparib. Niraparib induced greater PARP–DNA binding than olaparib, and veliparib was the least effective enhancer of PARP–DNA binding at concentrations that maximally inhibited PARP enzymatic activity. PARP–DNA binding was detected at pharmacologically relevant concentrations and correlated with the cytotoxicity of each agent *in vitro*. *In vivo*, enhanced PARP–DNA binding did not correlate with better anti-tumor activity but resulted in increased toxicity (22). The significance of differential PARP–DNA binding on efficacy and tolerability requires further investigation in the context of different tumor types and different PARP inhibitor and chemotherapy regimens. The complex role of PARPs in cellular homeostasis, including DNA repair, highlights the need to evaluate PARP inhibitors for modulating other biological functions of PARPs.

## Future Challenges of Parp Inhibitor Development

Clinical evaluation of the pharmacodynamic (PD) activity of PARP inhibitors has focused primarily on measuring inhibition of *ex vivo* enzymatic activity or PAR incorporation in tumor tissues and peripheral blood mononuclear cells (PBMCs). In a Phase 0 clinical trial, the National Cancer Institute and Abbott Laboratories validated a sandwich immunoassay to evaluate the PD response of veliparib during clinical development (23–25). The immunoassay measured changes in PARylated substrates collected from peripheral blood and tumor biopsy samples. While PD evaluations have demonstrated target engagement by veliparib and other PARP inhibitors, it is currently unclear what level of PARP inhibition is required to translate into a clinical response. In the case of olaparib, patients with BRCA-deficient ovarian or breast cancer demonstrated maximal PARP inhibition in PBMCs at doses greater than 60 mg BID olaparib capsules; however, dose-dependent anti-tumor activity was observed at higher doses of 100 and 400 mg BID olaparib capsules (26–28).

Several factors may contribute to the lack of a clear relationship between PARP inhibition and clinical activity. Exploratory analysis of olaparib pharmacokinetic (PK)/PD data suggested that sustaining unbound steady-state trough concentrations above the IC_90_ for PARP inhibition affords better clinical efficacy.^1^ These results correlated with *in vivo* PK/PD modeling of mouse tumor xenograft data that demonstrated a marked increase in DNA SSBs when PAR levels were decreased by more than 90%, and exceeding this threshold improved the anti-tumor activity of olaparib in BRCA-deficient tumors. A simulation of unbound steady-state trough concentrations in patients receiving 100, 200, and 400 mg BID olaparib capsules indicated that patients receiving 400 mg BID achieved steady-state trough concentrations exceeding the IC_90_ value for PARP inhibition. Other potential reasons for lack of a PK/PD relationship include off-target effects of PARP inhibitors or variability in PK data. Another possibility is that the cytotoxicity of PARP inhibitors may involve other mechanisms of action.

To date, investigation of the mechanisms of resistance to PARP inhibitor anti-tumor effects has been limited (29, 30). Potential mechanisms of resistance to PARP inhibitors may involve restoration of HR or modulation of PARP itself. One potential mechanism was demonstrated in the Capan-1 human metastatic pancreatic adenocarcinoma cell line, which lacks a wild type copy of *BRCA* while harboring a 6174delT mutant *BRCA* allele. This mutation causes a frameshift in the normal open reading frame (ORF), resulting in expression of truncated BRCA protein and a deficiency in HR (31, 32). Analysis of Capan-1 clones resistant to PARP inhibitors showed that additional mutations (i.e., deletion, insertion, or deletion/insertion) within *BRCA* in these cells rectified the 6174delT frameshift mutation and restored *BRCA2* normal ORF and BRCA function. Additional evidence that at least a partial restoration of HR can lead to resistance to PARP inhibitors include secondary mutations in the *BRCA* gene, restoring expression of wild type BRCA protein in patients (33) and somatic mutation of *TP53BP1* (34, 35).

In addition to restoration of HR, studies have also correlated resistance to PARP inhibitors with PARP itself and PD markers such as γ–H2AX (36, 37). In an *in vivo* study, responsiveness of mice bearing TC-71 Ewing sarcoma tumors to a combination of talazoparib and temozolamide was correlated with decreased levels of total or cleaved PARP and increases in γ–H2AX; however, tumors that were resistant to the combination treatment were shown to have some cleaved PARP but no decrease in total or cleaved PARP, or increases in γ–H2AX (38). Although the status of the genes involved in HR was not evaluated in tumors tested in this study, these results suggest that another potential mechanism of resistance to anti-tumor effects of PARP inhibitors may involve regulation of PARP itself.

The most concerning potential adverse reactions associated with PARP inhibition are myelodysplastic syndrome and acute myeloid leukemia (MDS/AML), especially in patients harboring a germline *BRCA* mutation. BRCA1 is critically involved with the Fanconi anemia proteins in repairing DNA damage, whereas BRCA2 is itself a Fanconi anemia protein. Biallelic mutations of *BRCA2* are linked to Fanconi’s anemia, a genetic disorder characterized by congenital abnormalities and a profound increase in cancer predisposition, namely AML (39, 40). The U.S. Package Insert for Lynparza (olaparib) contains the following warning for the development of MDS/AML: MDS/AML have been confirmed in 6 out of 298 (2%) patients enrolled in a single arm trial of Lynparza monotherapy, in patients with deleterious or suspected deleterious germline *BRCA-*mutated advanced cancers^2^. In a randomized placebo controlled trial, MDS/AML occurred in 3 out of 136 (2%) patients with advanced ovarian cancer treated with Lynparza. Overall, MDS/AML were reported in 22 of 2,618 (<1%) patients treated with Lynparza. The majority of MDS/AML cases (17 of 22 cases) were fatal, and the duration of therapy with Lynparza in patients who developed secondary MDS/cancer-therapy related AML varied from <6 months to >2 years. All patients had previous chemotherapy with platinum agents and/or other DNA damaging agents. The addition of further DNA damage induced by chemotherapy or other environmental factors, coupled with enhanced impairment of a compensatory repair pathway by means of PARP inhibition, may prime patients with germline DNA repair deficiencies for the development of MDS/AML. Monitoring of complete blood counts and perhaps PBMCs for micronuclei is warranted for patients receiving PARP inhibitors, and further investigations should be performed for prolonged hematologic toxicity (see text footnote 2).

## Conclusion

Notwithstanding the current knowledge regarding the biological role of PARP and its demonstrated clinical benefit in cancers with germline *BRCA* mutations, future studies are needed to improve the therapeutic potential of PARP inhibitors. For example, better understanding of the contribution of the various mechanisms of action *in vivo*, in the context of different PARP inhibitors and different tumor types, together with better understanding of mechanisms of resistance will aid in improving the therapeutic potential of this class of drugs by optimizing patient selection (e.g., based on baseline or PARP inhibitor-mediated changes in HRD profile) or optimizing selection of therapeutic agents in combination clinical trials by targeting separate mechanisms of drug resistance. Additionally, studies are needed to identify predictive biomarkers and to develop validated, diagnostic tests to extend the therapeutic landscape of PARP inhibitors beyond *BRCA*-mutated tumors (41–43).

## Author Note

This article reflects the views of the authors and should not be construed to represent FDA’s views and policies.

## Conflict of Interest Statement

The authors declare that the research was conducted in the absence of any commercial or financial relationships that could be construed as a potential conflict of interest.

## Footnotes

^1^Pharmacology/Toxicology NDA review: olaparib 2014. Available from: http://www.accessdata.fda.gov/drugsatfda_docs/nda/2014/206162Orig1s000PharmR.pdf

^2^LYNPARZA™(olaparib) label: Available from http://www.accessdata.fda.gov/scripts/cder/drugsatfda/
